# Progress with the *Southern African Journal of Infectious Diseases*: Where are we in 2021?

**DOI:** 10.4102/sajid.v36i1.352

**Published:** 2021-12-17

**Authors:** Charles Feldman

**Affiliations:** 1 Department of Internal Medicine, Faculty of Health Sciences, University of the Witwatersrand, Johannesburg, South Africa

The *Southern African Journal of Infectious Diseases* (SAJID) had undergone numerous changes over the years, largely because of evolutionary changes in journal publication practices. Amongst these changes were the following:

- Modernisation of the look of the journal.
- Placement of the journal on an open access platform.
- Changing the name of the journal from the *Southern African Journal of Epidemiology and Infection* to the *Southern African Journal of Infectious Diseases*.
- The journal was placed on a sound financial footing, with the introduction of ‘page fees’.

Despite the introduction of costs for publishing in the journal, the journal still continues to elicit numerous manuscript submissions, and pleasingly we have also seen an increase in submissions from sub-Saharan Africa, and beyond that including some submissions from the Middle East and even Europe.

The journal remains the ‘mouthpiece’ of the Federation of Infectious Diseases Societies of Southern Africa (FIDSSA), an umbrella group of most infectious diseases societies in South Africa, and has been Department of Higher Education and Training (DHET) accredited for many years, both by way of being on the DHET SA list, and since 2021, by being on the Directory of Open Access Journals (DOAJ). What this means, amongst other considerations, is that articles published in the journal accrue governmental subsidy for the universities of the authors.

Remarkably, all articles published in the journal are included in the African Index Medicus, DOAJ, Ebsco Host, Gale, Cengage Learning, Google Scholar and Hinari.

The major next step that all potential authors hoped for was the indexing of the journal in PubMed Central. We are thrilled to announce, officially, as many of you may already know that the journal is now indexed in PubMed Central and also included in the Web of Science Other Coverage, Emerging Sources Citation Index (ESCI). As the Pubmed Central website indicates, for journals in the medical and life sciences, being indexed in PubMed is a high priority goal. Clearly the benefits are that it significantly increases the trust in the journals processes and its commitment to science, so that it becomes easier to attract high quality submissions. It also increases the journal’s visibility, leading to an increased reach and exposure of the journal with the hope of an increase in citations and eventually achieving an impact factor. With regard to indexing, the journal is highly appreciative of the role of all our loyal reviewers, the editorial board members and our publishers, AOSIS (Pty) Ltd, for their great professionalism.

However, we cannot stop here and rest on our laurels. Recent activity that has aimed to achieve even greater heights for the journal include the following:

- Revision of the editorial board, in line with the common recommendations.
- The introduction of an improved process of facilitating the award of continuing professional development (CPD) points for both authors and reviewers for the journal.
- We need to strive further to increase our citations and therefore be granted an impact factor.

Furthermore, it is time for the journal to continue succession planning for the next editor-in-chief (EIC). I had hoped that during my tenure as EIC I would at least be able to get the journal to the point where we are now. Having got there, I think it is therefore time for the appointment of a new leader, who as the saying goes, could be the new broom that sweeps clean, and who could lead the journal to even greater heights.

I thank you for your confidence in me over all these years.

